# Combined chemoradiation of cisplatin versus carboplatin in cervical carcinoma: a single institution experience from Thailand

**DOI:** 10.1186/s12885-016-2558-9

**Published:** 2016-07-19

**Authors:** Ekkasit Tharavichitkul, Vicharn Lorvidhaya, Pimkhuan Kamnerdsupaphon, Vimol Sukthomya, Somvilai Chakrabandhu, Pitchayaponne Klunklin, Wimrak Onchan, Bongkoch Supawongwattana, Nantaka Pukanhaphan, Razvan Galalae, Imjai Chitapanarux

**Affiliations:** The Division of Therapeutic Radiology and Oncology, Department of Radiology, Faculty of Medicine, Chiang Mai University, Chiang Mai, Thailand; Faculty of Medicine, Christian-Albrechts-University, Kiel, Germany; Department of Radiooncology, Evangelical Clinics, Gelsenkirchen, Germany

**Keywords:** Cervical carcinoma, Chemo-radiation, Cisplatin, Carboplatin

## Abstract

**Background:**

To report the results of combined chemoradiation (CCRT) with cisplatin versus carboplatin in locally advanced cervical carcinoma.

**Methods:**

From 2009 to 2013, 255 patients with stage IIB-IVA cervical carcinoma, according to FIGO staging were prospectively assigned to be treated with pelvic radiotherapy followed by brachytherapy given concurrently with cisplatin or carboplatin in the treatment of locally advanced cervical cancer. Treatment outcomes and toxicitiy were evaluated.

**Results:**

Two-hundred and thirteen patients could be evaluated. At a median follow-up time of 43 months (6–69 months), the 3-year local control, disease-free survival, metastasis-free survival and overall survival rates were 93, 80.8, 85.0 and 87.3 %, respectively. No statistical difference in terms of local control, disease-free survival, metastasis-free survival and overall survival rates between cisplatin and carboplatin treatments was observed in this study. Eighty-six percents of the patients in the carboplatin group could receive more than 4 cycles, while there were only 72 % in the cisplatin group who completed more than 4 cycles (*p* = 0. 02). In terms of acute toxicity, cisplatin caused significantly more anemia (*p* = 0.026), neutropenia (*p* = 0. 044) and nephrotoxicity (*p* = 0. 031) than carboplatin. No difference in late toxicity was observed in this study.

**Conclusion:**

Carboplatin yielded comparable results to cisplatin in concurrent chemo-radiation for locally advanced cervical cancer. In addition, carboplatin was associated with a better compliance rate and was associated with less of anemia, neutropenia and nephrotoxicity.

## Backgrounds

Cervical carcinoma is one of the most frequent cancer entities in Northern Thailand. According to the report of Kamnerdsupaphon et al. the age-standardized incidence rate22.7 per 100,000 and there were 234 new cases of cervix cancer diagnosed in 2005 and was one of the three most common cancers in female. Moreover, it ranked second after breast cancer [1]. The treatment options of cervical cancer are composed of surgery, radiotherapy and/or chemotherapy according to stage and performance status of patients. For locally advanced disease, pelvic radiation and brachytherapy given concurrently with a platinum-based regimen is the standard treatment [2–4].

Weekly cisplatin is the most commonly used regimen for chemoradiation. However, it may cause acute toxicities, including nephrotoxicity and ototoxicity [5]. The comparative data between cisplatin and carboplatin were generally assessed in metastatic disease and mostly in phase II or retrospective studies [6–8]. In locally advanced cervical cancer, no prospective study has compared outcomes and treatment related toxicities between the two types of chemotherapy. One retrospective study on cisplatin versus carboplatin for concurrent chemoradiation for locally advanced cervical cancer reported carboplatin did not showed benefits over cisplatin [9]. To our knowledge, there were many published studies that reported the experiences of concurrent carboplatin [10–15]. In our center, carboplatin has been used for patients with poor functional status or age >70 or any age with poor creatinine clearance. The question of using carboplatin in good performance status needs to be answered. We performed this observational study to evaluate prospectively the disease-related outcomes and therapy –related toxicity of cisplatin versus carboplatin in concurrent chemoradiation for patients with locally advanced cervical cancer.

## Methods

### Patients

Two-hundred and fifty five patients with biopsy proven cervical cancer were included in this study from February 2009 to February 2013. All enrolled cervical cancer patients were classified as IIB-IVA by FIGO clinical staging, were 18–70 years old at study entry and had a Karnofsky performance status > 70 %. All patients had normal laboratory results with hemoglobin (Hb) ≥10 g/dl, white blood count (WBC) ≥3000 cells/mm^3^, platelet count ≥ 100,000 cells/mm^3^, serum creatinine clearance ≥50 ml/min and normal liver function tests.

Patients with a severe co-morbidity, pregnancy, previous radiotherapy, distant metastases at diagnosis or history of allergies were excluded from the study.

This study was approved by the Ethic committee of faculty of medicine, Chiang Mai University with the study code of RAD-08-12-12A-13. This study was performed in accordance with the principles of human clinical trials and the Helsinki Declaration (1975 edition and 2000 revised edition). All of the patients signed informed consent before treatment.

#### Radiotherapy techniques

Radiotherapy for cervical cancer in our institution is performed in concordance with the publication by Lorvidhaya et al. [16].

#### Whole pelvic radiotherapy (WPRT)

All patients received WPRT to the primary tumor and pelvic lymph nodes by conventional radiotherapy to the total dose of 50 Gy in 25 fractions. After 44 Gy, central shielding was conventionally added to reduce the bladder and rectal dose. After 50 Gy, parametrial boost was performed to a cumulative dose of 56 Gy according to our institutional protocol.

#### Intracavitary Brachytherapy (ICBT)

ICBT was used in all patients. The tandem/ovoids (Fletcher-Williamson “Asia-Pacific” model) or tandem/cylinder (in case of more than one-third vaginal involvement) were used. Four applications were performed in all patients. The first BT application was generally planned after the fourth week of WPRT with 1–2 fractions per week. The total dose of 24–28 Gy in 4 fractions was prescribed.

#### Combined chemo-radiation (CCRT)

**Fig. 1:**
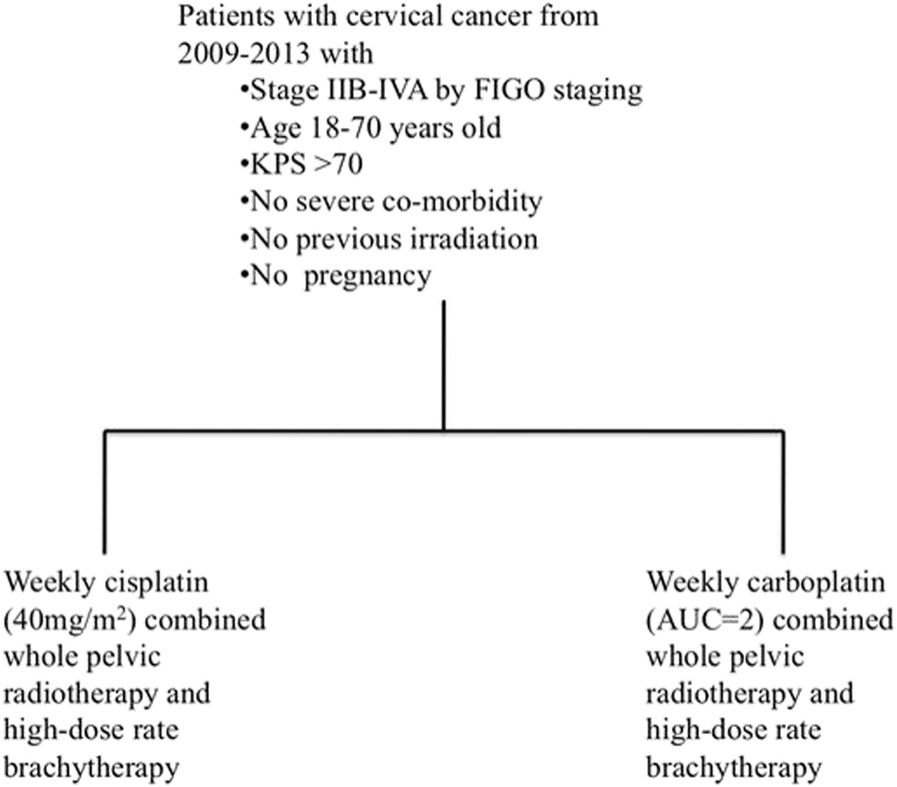
Treatment schema

All enrolled patients were assigned to receive cisplatin or carboplatin after study enrollment. CCRT with weekly cisplatin at a dose of 40 mg/m^2^ or weekly carboplatin at a dose of area under curve (AUC) equal to 2 for a maximum of six courses was given to all patients with sufficient kidney and bone marrow function during the EBRT. For the dosing of carboplatin, glomerular filtration rate (GFR) was calculated from serum creatinine, age and body weight and the dose of AUC 2 was calculated from the GFR by using Calvert’s formula [17]. The maximum dose of chemotherapy for each cycle was 70 mg of cisplatin and 200 mg of carboplatin. Complete blood counts and renal function tests (serum blood urea nitrogen and creatinine) were evaluated weekly before consideration of chemotherapy. The dose of chemotherapy was modified according to a weekly assessment of creatinine clearance prior to each applied dose. Chemotherapy was interrupted when creatinine clearance was less than 40 ml/min for cisplatin and it was stopped when creatinine clearance was less than 30 ng/ml (Fig. 1).

#### Study end point

The primary end point was overall survival rate. Other treatment results and toxicies were evacuate as secondary end point. All events were measured from the date of randomization to the date of their occurrence or the the date of the last follow-up visit. Toxicity were graded according to the Common Terminology Criterias of Adverse Events (CTCAE) version 3.0 [18].

#### Follow-up program

After treatment was completed, patients were appointed to visits including vaginal examination (PV exam) in a follow-up program. The follow-up schedule included visits every 3–4 months in the first 2 years after treatment, every 6 months in the 3^rd^ to 5^th^ year and then annually after the 5^th^ year. A vaginal examination was performed to evaluate the disease status according to the World Health Organization (WHO) criteria [19]. Further investigations to identify disease progression were performed when indicated by clinical findings. Late toxicities were evaluated according to the Radiation Therapy Oncology Group/European Organization of Research and Treatment of Cancer (RTOG/EORTC) late toxicity criteria [20]. CTCAE version 3.0 was only used to evaluate vaginal obstruction.

#### Statistical analysis

Study enrollment was planned for 5 years and performed from 2009–2013. Overall survival (OS), metastasis-free survival (MFS), disease-free survival (DFS) and local control (LC) rates were evaluated using Kaplan-Meier estimates and compared using the log-rank test. Clinical and patient factors were compared using Pearson’s chi-squared, Mann–Whitney-U or Independent-Samples *T* test as appropriate. All statistical analyses were performed using the SPSS version 17.00 (IBM company software, Chicago, Illinois, USA).

## Results

### Patient characteristics

Forty-two patients were excluded from analysis process due to incomplete treatment schedule (31 patients) and addition of neoadjuvnat chemotherapy (11 patients). Thus, 213 patients could be evaluated. One hundred thirty seven patients received weekly cisplatin and 76 patients weekly carboplatin. In the whole group, the mean age was 52.4 years (21–70 years). The mean maximal diameter of primary lesion was 4.4 cm (2–9 cm). Stage IIB was the most common in patients (134 patients). 180 patients (84.5 %) had squamous cell carcinoma (SCCA) and the most common grade was moderately differentiated (58.7 %). The median follow-up time was 43 months (6–69 months) and the mean total treatment time was 56.5 days (34–173 days). All patient characteristics were shown in Table 1.

**Table 1:** Patient characteristics by study groups

Parameters	cisplatin (*N* = 137)	carboplatin (*N* = 76)	*P*-value
Age (years)	51.6 (21–67)	53.8 (35–70)	0.051
Maximal diameter of primary tumor(cm)	4.5 (2–8)	4.3(2–9)	0.4
Stage			0.09
IIB	91(66.4 %)	43(56.6 %)	
IIIA	0	2(2.6 %)	
IIIB	44(32.1 %)	31(40.8 %)	
IVA	2(1.5 %)	0	
Pelvic Lymph nodes			0.63
No	72(52.6 %)	45(59.2 %)	
Yes	14(10.2 %)	6(7.9 %)	
Unknown	51(37.2 %)	25(32.9 %)	
Histology			0.4
SCCA	114(83.2 %)	66(86.8 %)	
ACA	21(15.3 %)	9(11.8 %)	
Others	2(1.5 %)	1(1.3 %)	
Histologic grade			0.66
1	18(14.8 %)	9(13.4 %)	
2	78(63.9 %)	47(70.1 %)	
3	26(21.3 %)	11(16.4 %)	
Cycles			0.02
Up to 4 cycles	38 (27.7 %)	10(13.2 %)	
5 to 6 cycles	99(72.3 %)	66(86.8 %)	
Total treatment time (days)	56.8 (34–114)	56(43–173)	0.39
Median follow-up time (months)	39.8(6–69)	40.6(6–69)	0.76

*Note*: *SCCA* squamous cell carcinoma, *ACA* adenocarcinoma

### Treatment results

**Fig. 2:**
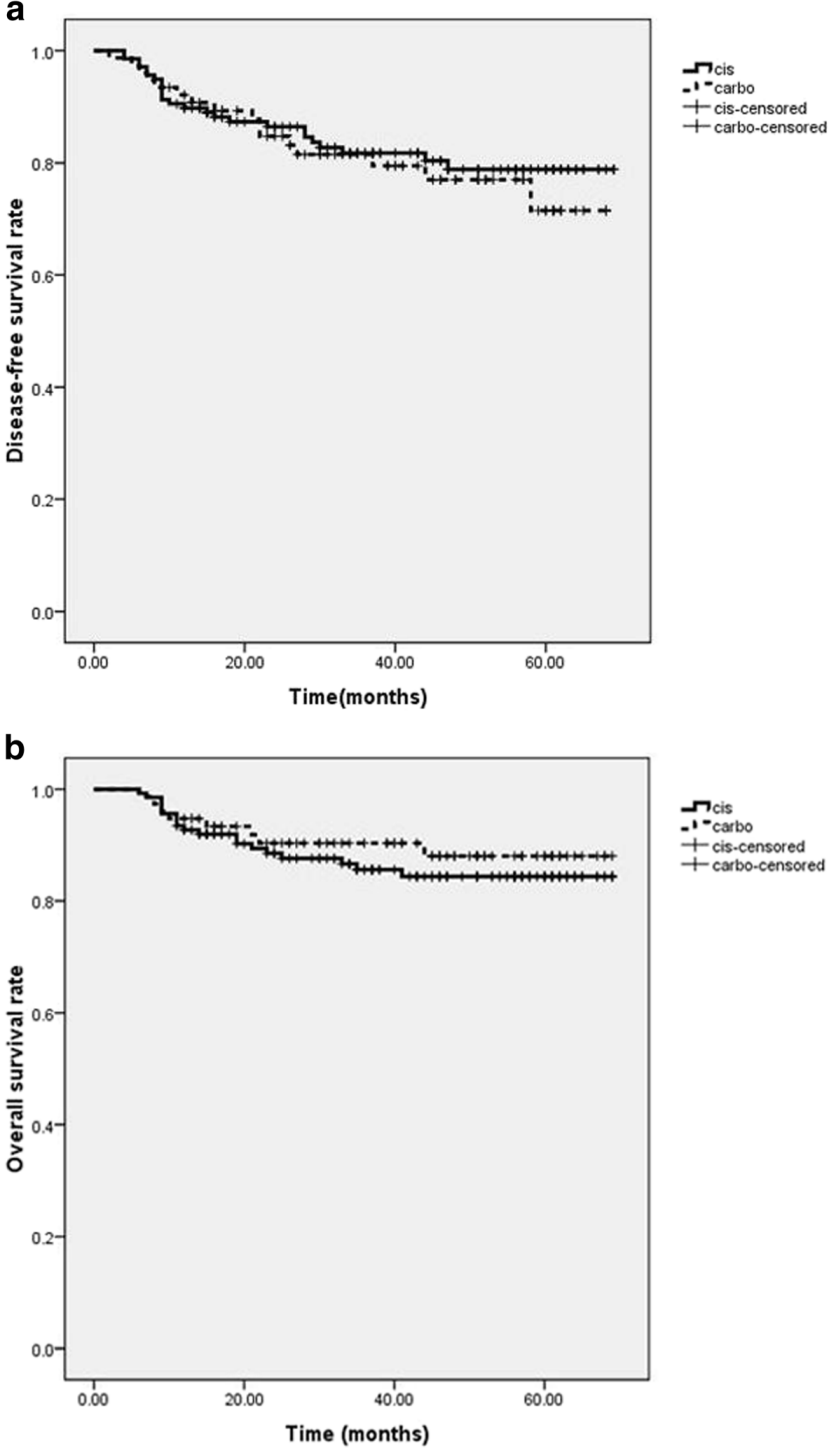
Kaplan-Meier curves showing (**a**) disease-free survival rate and (**b**) overall survival rates of cisplatin (*continuous line*) versus carboplatin (*dotted line*)

The 3-year local control, disease-free survival, metastasis-free survival and overall survival rates were 93 %, 80.8, 85 and 87.3 %, respectively. 33 patients (15.5 %) developed distant metastasis and 18 patients (8.4 %) developed lymph node metastases. No statistical significance in terms of local control, disease-free survival, metastasis-free survival and overall survival rates was observed between the cisplatin and carboplatin cohorts. All data are detailed in Table 2 and Fig. 2.

**Table 2:** The 3-year local control, disease-free survival, metastasis-free survivaland overall survival rates of cisplatin versus carboplatin

Parameters	Cisplatin (*N* = 137)	Carboplatin (*N* = 76)	*p*-value
Local control	94.9 %	89.5 %	0.13
Disease-free survival	81.8 %	78.9 %	0.62
Metastasis-free survival	83.9 %	86.8 %	0.56
Overall survival	86.1 %	89.5 %	0.48

**Fig. 3:**
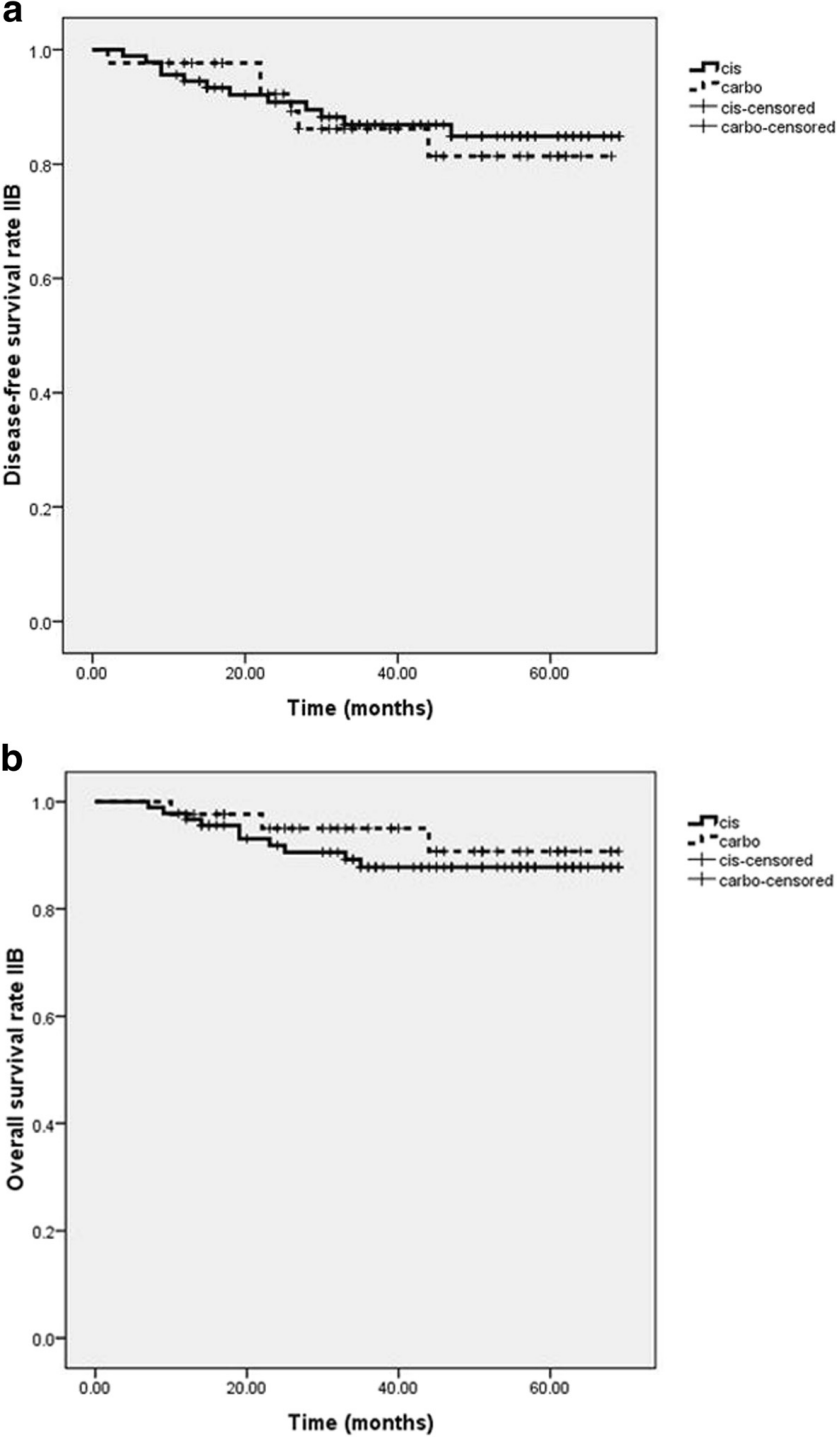
Kaplan-Meier curves showing (**a**) disease-free survival rate and (**b**) overall survival rates of cisplatin (*continuous line*) versus carboplatin (*dotted line*) in stage IIB of cervical carcinoma

**Fig. 4:**
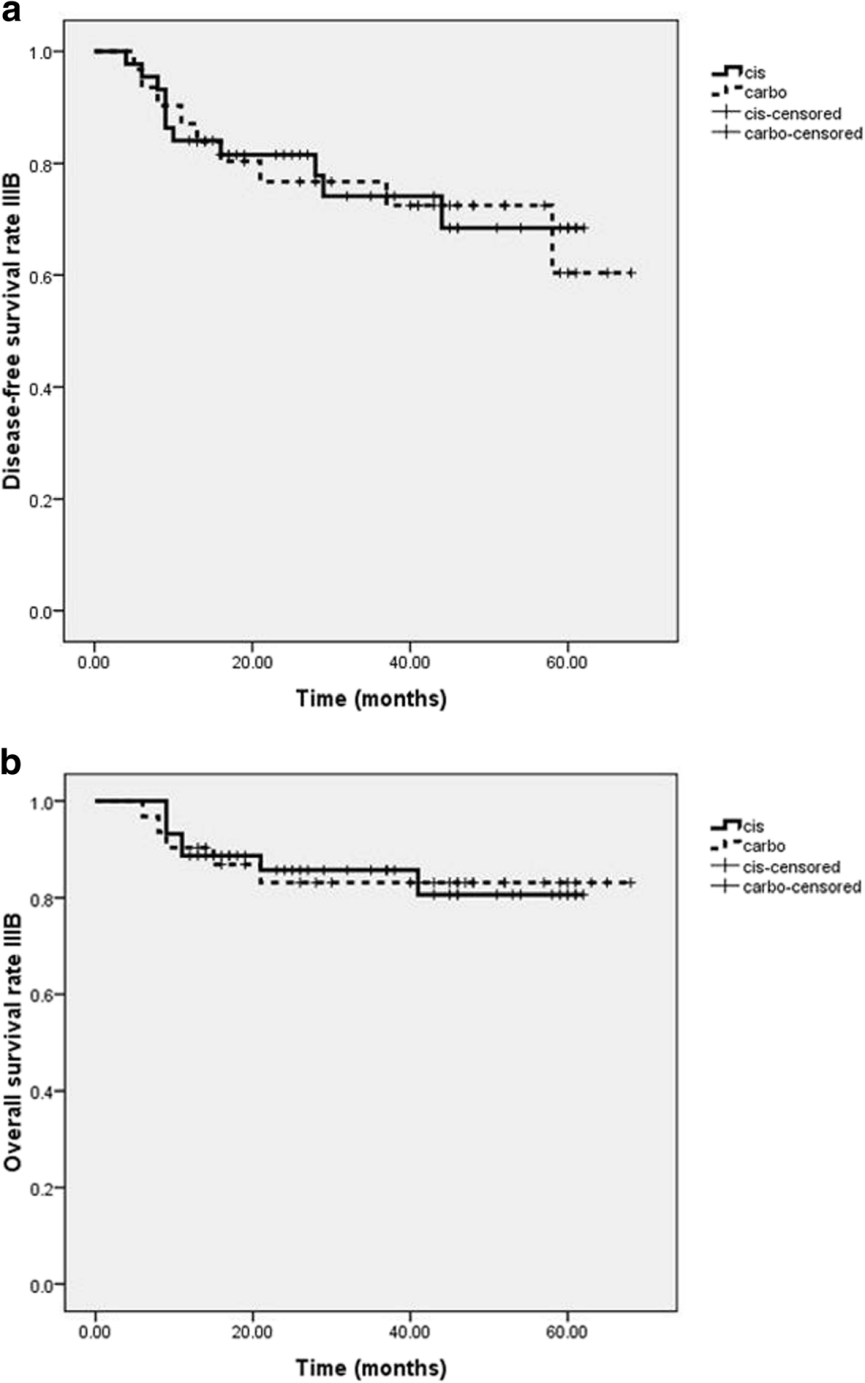
Kaplan-Meier curves showing (**a**) disease-free survival rate and (**b**) overall survival rates of cisplatin (*continuous line*) versus carboplatin (*dotted line*) in stage IIIB of cervical carcinoma

Analyses were performed by stratification on stage IIB (135 patients) and stage IIIB (74 patients) which composed the largest populations in these groups. In stage IIB, the local control, disease-free survival, metastasis-free survival and overall survival rates were 96.3, 86.6, 88.1, and 90.3 %, respectively. In stage IIIB, the local control, disease-free survival, metastasis-free survival and overall survival rates were 89.3, 73.3, 80 and 84 %, respectively. No statistically significant difference between both study groups in terms of local control, disease-free survival and overall survival rates could be identified stratified by stage. All data of stage IIB and IIIB are shown in Table 3 and Figs. 3 and 4.

**Table 3:** The 3- year local control rate, disease-free survival rate, metastasis-free survival and overall survival rate of cisplatin versus carboplatin according to stage

Stage IIB	Cisplatin (*N* = 137)	Carboplatin (*N* = 76)	*p*-value
Local control	96.7 %	95.3 %	0.68
Disease-free survival	86.8 %	86 %	0.78
Metastasis-free survival	87.9 %	88.4 %	0.98
Overall survival	89 %	93 %	0.5
Stage IIIB	Cisplatin (*N* = 137)	Carboplatin (*N* = 76)	*p*-value
Local control	93.2 %	83.9 %	0.22
Disease-free survival	75 %	71 %	0.88
Metastasis-free survival	77.3 %	83.9 %	0.44
Overall survival	84.1 %	84 %	0.99

### Toxicity profiles

The most common toxicities were anemia (18.8 % or 40 patients) for acute toxicity and gastrointestinal side effects (14.6 % or 31 patients) for late toxicity. In terms of acute toxicity, patients who received cisplatin developed higher rates of anemia, neutropenia and renal toxicity than the patients in the carboplatin group. No difference in thrombocytopenia, radiation dermatitis, proctitis or cystitis was observed. All data of acute toxicity are shown in Table 4.

**Table 4:** Acute toxicity of cisplatin versus carboplatin

Acute toxicity	cisplatin (*N* = 137)	carboplatin (*N* = 76)	*P*-value
Anemia			0.026
Grade 0	87(63.5 %)	57(75.0 %)	
Grade 1	19(13.9 %)	10(13.2 %)	
Grade 2	28(20.7 %)	5(6.6 %)	
Grade 3	3(2.2 %)	4(5.3 %)	
Neutropenia			0.044
Grade 0	84(61.3 %)	57(75 %)	
Grade 1	25(18.2 %)	14(18.4 %)	
Grade 2	25(18.2 %)	5(6.6 %)	
Grade 3	3(2.2 %)	0	
Thrombocytopenia			0.392
Grade 0	128(93.4 %)	72(94.7 %)	
Grade 1	4(2.9 %)	0	
Grade 2	4(2.9 %)	4(5.3 %)	
Grade 3	1(0.7 %)	0	
Renal toxicity			0.031
Grade 0	112(81.8 %)	73(96.5 %)	
Grade 1	8(5.8 %)	1(1.3 %)	
Grade 2	10(7.3 %)	1(1.3 %)	
Grade 3	7(5.1 %)	1(1.3 %)	
Skin toxicity			0.45
Grade 0	139(94.9 %)	75(98.7 %)	
Grade 1	4(2.9 %)	1(1.3 %)	
Grade 2	3(2.2 %)	0	
Proctitis			0.94
Grade 0	122(89.1 %)	67(88.2 %)	
Grade 1	9(6.6 %)	6(7.9 %)	
Grade 2	6(4.4 %)	3(3.9 %)	
Cystitis			0.066
Grade 0	130(94.9 %)	67(88.2 %)	
Grade 1	7(5.1 %)	7(9.2 %)	
Grade 2	0	2(2.6 %)	

In terms of late toxicity, no statistically significant difference between the cisplatin and carboplatin groups for gastrointestinal, genitourinary, dermatological or vaginal toxicity was found. All data of late toxicity stratified by study group are shown in Table 5.

**Table 5:** Late toxicity profiles of cisplatin versus carboplatin

Late toxicity	cisplatin (*N* = 137)	carboplatin (*N* = 76)	*p*-value
Chronic GI			0.331
Grade 0	103(75.2 %)	63(82.9 %)	
Grade 1	13(9.5 %)	3(3.9 %)	
Grade 2	15(10.9 %)	5(6.6 %)	
Grade 3	2(1.5 %)	3(3.9 %)	
Grade 4	4(2.9 %)	2(2.6 %)	
Chronic GU			0.206
Grade 0	122(89.1 %)	69(90.8 %)	
Grade 1	6(4.4 %)	1(1.3 %)	
Grade 2	6(4.4 %)	1(1.3 %)	
Grade 3	1(0.7 %)	3(3.9 %)	
Grade 4	2(1.5 %)	2(2.6 %)	
Chronic skin			1.00
Grade 0	135(98.5 %)	75(98.7 %)	
Grade 1	1(0.7 %)	0	
Grade 2	1(0.7 %)	1(1.3 %)	
Subcutaneous tissue			0.849
Grade 0	117(85.4 %)	65(85.5 %)	
Grade 1	13(9.5 %)	6(7.9 %)	
Grade 2	7(5.1 %)	5(6.6 %)	
Vaginal obstruction			0.281
Grade 0	101(73.7 %)	58(76.3 %)	
Grade 1	17(12.4 %)	9(11.8 %)	
Grade 2	18(13.1 %)	6(7.9 %)	
Grade 3	1(0.7 %)	3(3.9 %)	

## Discussion

The present study reports outcomes following CCRT using carboplatin in comparison to cisplatin for locally advanced carcinoma of cervix uteri in good general condition. Our data show comparable results of carboplatin versus cisplatin in CCRT in terms of local control, disease-free survival, metastasis-free survival and overall survival rates with *p*-value of 0.13, 0.62, 0.56 and 0.48, respectively. The present results are in concordance with the studies of Nam et al. and Au-Yeung et al. in terms of survival rates [9, 21]. In stage IIB, local control, disease-free survival, metastasis-free survival and overall survival rates between group I and group II were not statistically significant. In stage IIIB, no statistical significance in all endpoints could be observed as well.

In addition, the patient characteristics of both groups were well balanced without statistically significant differences in age, stage, maximal diameter, grade, histology, follow-up time and total treatment time although the mean age of group II (carboplatin) was slightly higher than group I (cisplatin) (53.8 years vs. 51.6 years; *p* = 0.051). Group II patients had a higher compliance rate (received > 4 cycles) than group I (86.8 % vs. 72.3 %; *p* = 0.02) and this finding matched the results of Nam et al. who showed that the numbers of received cycles of carboRT was higher than cisRT (7.5 versus 6; *p* < 0.001) [21]. The main difference which was found in the present study is the acute toxicity. Cisplatin significantly caused more anemia (*p* = 0.026), neutropenia (*p* = 0.044) and nephrotoxicity (*p* = 0.031) than carboplatin while other parameters were equal. However, no statistical significance between the two groups in terms of late dermatotoxicities, gastrointestinal toxicities, genitourinary toxicities and vaginal toxicities were observed.

Carboplatin, either in combination or as a single-agent, may offer advantages in patients aged >75 years and in those with decreased performance status [22]. Many studies reported the use of weekly carboplatin in combination with WPRT in the treatment of locally advanced cervical cancer. When we compared the carboplatin group with other studies, it showed comparable results (Table 6).

**Table 6:** Studies of carboplatin in CCRT for locally advanced cervical cancer

Studies	N	Stage	Treatment results	Late Toxicities
Cetina et al. [11]	85 (high risk of poor renal dysfunction)	IB2 4.7 %	OS 81 %	-
		IIA 8.2 %		
		IIB 41.1 %		
		IIIA 4.7 %		
		IIIB 38.8 %		
		IVA 2.5 %		
Cetina et al. [12]	59 (elderly patients)	IB2 8.4 %	30-months OS 63 %	-
		IIA 13.5 %		
		IIB 52.5 %		
		IIIA 3.3 %		
		IIIB 18.6 %		
Katanyoo et al. [13]	148	IIB 50.7 %	2-years PFS 75.1 %	Grade 3–4 GI 10.1 %
		IIIB 48.0 %	5-years PFS 63 %	Grade 3–4 GU 0.7 %
		IVA 1.3 %	2-years OS 81.9 %	
			5-years OS 63.5 %	
Sangkittipaiboon et al. [14]	105	IIB 83	5-years DFS 52.38 %	Grade 3–4 GI 3.2 %
		III, 19	5-years OS 56.19 %	Grade 3–4 GU 0 %
		IVA 3		
Our study	76	IIB 56.6 %	3-years DFS 78.9 %	Grade 3–4 GI 6.5 %
		IIIA 2.6 %	3-years OS 89.5 %	Grade 3–4 GU 6.5 %
		IIIB 40.8 %		

*Note*: *PFS* progression-free survival, *DFS* Disease-free survival, *OS* overall survival, *GI* gastrointestinal, *GU* genitourinary

This study has some limitations. Firstly, this study is observational study. This study was firstly designed as non-inferiority study. The sample size for this study is determined to compare the 5-year survival rate and at least 223 patients in each arm were required to provide at least 80 % power to be able to detect this difference in 5-year survival rate between the study arms for a two-sided test with alpha = 0.05. Unfortunately, the enrollment has problem due to poor accrual. Secondly, 42 patients had to be excluded from analysis. Eleven patients received neo-adjuvant chemotherapy and 31 patients had incomplete treatment.

Despite the mentioned limitations, this study showed similar results in terms of local control, disease-free survival, metastasis-free survival and overall survival rate in both study arms along with a better compliance rate and lower acute toxicity in terms of neutropenia and nephrotoxicity in the carboplatin cohort. This study results support the use of carboplatin in CCRT for locally advanced carcinoma of cervix uteri in patients with good general conditions. Moreover, the administration of carboplatin is generally easier than cisplatin. In our routine practice, the whole chemotherapy process per cycle lasts three hours for carboplatin versus six hours for cisplatin. In the context of a high-volume, high-workload institute, carboplatin-based CCRT is easier to manage.

## Conclusions

The use of carboplatin in combination with radiation therapy is comparable to cisplatin in terms of treatment outcomes. In addition, the compliance in the carboplatin arm was better and the observed acute toxicity in terms of anemia, neutropenia and neprotoxicity was lower in the carboplatin versus cisplatin group. The present study results encourage the use of carboplatin in the treatment of locally advanced cervix carcinoma in patients with good general condition.

## Abbreviations

ACA, adenocarcinoma; AUC, area under curve; CCRT, combined chemoradiation; CTCAE, common terminology criterias of adverse events; DFS, disease-free survival; FIGO, international federation of gynecology and obstetrics; GFR, glomerular filtration rate; GI, gastrointestinal; GU, genitourinary; ICBT, intracavitary brachytherapy; OS, overall survival; PFS, progression-free survival; RTOG/EORTC, Radiation Therapy Oncology Group/European Organization of Research and Treatment of Cancer; SCCA, squamous cell carcinoma; WPRT, whole pelvic radiotherapy
